# Ensemble Machine Learning Predicts Platinum Resistance in Ovarian Cancer Using Laboratory Data

**DOI:** 10.3390/cancers18081190

**Published:** 2026-04-08

**Authors:** Xueting Peng, Yangyang Zhang, Chaoyu Zhu, Weijie Chen, Xiaohua Wu, Fan Zhong, Qinhao Guo, Lei Liu

**Affiliations:** 1 Shanghai Institute of Infectious Disease and Biosecurity, Fudan University, Shanghai 200032, China; 23211020027@m.fudan.edu.cn; 2 Department of Gynecologic Oncology, Fudan University Shanghai Cancer Center, Shanghai 200032, China; 21301050051@m.fudan.edu.cn (Y.Z.); wu.xh@fudan.edu.cn (X.W.); 3 Intelligent Medicine Institute, Fudan University, Shanghai 200032, China; cyzhu18@fudan.edu.cn (C.Z.); weijie_chen@fudan.edu.cn (W.C.); zhongfan@fudan.edu.cn (F.Z.); 4 Shanghai Institute of Stem Cell Research and Clinical Translation, Shanghai 200120, China

**Keywords:** ovarian cancer, platinum resistance, machine learning, predictive model, routine laboratory data, ensemble learning, neoadjuvant chemotherapy

## Abstract

Objectives: Platinum resistance remains a critical bottleneck in ovarian cancer management, yet reliable pre-treatment predictive tools are lacking. Existing markers like the platinum-free interval are retrospective, while genomic profiling is often cost-prohibitive. This study aimed to develop an accessible, machine learning-based dynamic weighted fusion (DWF) model using routine laboratory data to provide bidirectional risk stratification, particularly to reliably rule out platinum resistance before treatment initiation. Methods: In this retrospective study (2019–2023), seventy baseline clinical features were collected to differentiate platinum-resistant from platinum-sensitive ovarian cancer patients. We developed a DWF framework that dynamically integrates the top-performing classifiers from a library of 168 algorithms (combining 14 feature selection and 12 machine learning methods). Class imbalance was addressed via oversampling, and model efficacy was evaluated using area under the curve (AUC), accuracy, sensitivity, and specificity. Results: The DWF model achieved a robust AUC of 0.760 (95% CI: 0.683–0.837), outperforming all individual base classifiers. Subgroup analysis demonstrated highly consistent overall discrimination across initial treatment strategies (AUC of 0.755 for primary debulking surgery and 0.761 for neoadjuvant chemotherapy). Feature interpretation highlighted that resistance is driven by synergistic dysregulation of systemic inflammation and hypercoagulability, rather than single biomarkers. Conclusions: The proposed DWF model effectively leverages low-cost, standardized clinical data to serve as a robust bidirectional stratification tool. Its exceptional ability to rule out resistance provides clinicians with the evidence-based confidence to proceed with standard therapies, while its high-risk alerts identify candidates for early therapeutic adjustments and enhanced surveillance in ovarian cancer care.

## Simple Summary

Ovarian cancer is a leading cause of death among women, largely because many patients are resistant to the standard platinum-based chemotherapy. Currently, doctors cannot predict this resistance until after the treatment has failed, delaying effective care. This study aimed to solve this problem by developing a low-cost, accessible prediction tool using artificial intelligence and routine laboratory data collected before treatment. We developed a new ensemble model that analyzes common laboratory tests, such as complete blood counts and liver and kidney function panels, to provide a comprehensive risk profile for each patient. Our model proved highly effective at “ruling out” resistance, giving doctors strong evidence to confidently proceed with standard chemotherapy for patients predicted to be sensitive. Simultaneously, it serves as an early-warning system for patients at high risk of resistance. This finding is significant because it offers a practical, non-invasive way to personalize therapy through bidirectional risk stratification, potentially improving survival rates without requiring expensive or complex genetic testing.

## 1. Introduction

Ovarian cancer represents a significant threat to women’s health globally, ranking as the eighth most common cancer among women worldwide [1]. In China, it is particularly devastating, standing as the leading cause of death among all gynecological malignancies [2,3]. The prognosis for patients remains poor, with a five-year survival rate of only 51.6% [4], underscoring the urgent need for improved therapeutic strategies.

The standard of first-line therapy for ovarian cancer involves primary debulking surgery (PDS) followed by platinum-based adjuvant chemotherapy [5]. For patients unsuitable for PDS, neoadjuvant chemotherapy (NAC) followed by interval debulking surgery is recommended [6]. However, individual responses to these treatments vary significantly. Approximately 20% of patients exhibit primary resistance to first-line therapy, leading to poor treatment outcomes [7]. Furthermore, 50% to 70% of patients who initially respond will eventually relapse due to acquired resistance, severely impacting their prognosis [8]. This highlights the limitations of a traditional “one-size-fits-all” chemotherapy approach and further emphasizes the need for strategies that can identify drug resistance earlier [9]. In particular, the ability to identify platinum resistance early could optimize personalized treatment and potentially extend patient survival, yet no feasible pre-treatment prediction technique currently exists [10].

Currently, platinum resistance in ovarian cancer is a retrospective diagnosis determined by the platinum-free interval (PFI), defined as the time from the last dose of platinum-based chemotherapy to disease recurrence [11]. Clinically, patients are stratified based on PFI into three categories: platinum-resistant (PFI < 6 months), partially platinum-sensitive (PFI 6–12 months), and fully platinum-sensitive (PFI > 12 months) [7]. However, reliance on these retrospective outcomes creates a lag in decision-making. Consequently, one of the central challenges in gynecologic oncology is the inability to accurately predict platinum sensitivity before treatment initiation. Early identification of potential platinum-resistant patients would enable clinicians to implement more proactive interventions. These could include intensifying the initial chemotherapy cycles, considering intraperitoneal chemotherapy, or exploring non-platinum-based agents with demonstrated potential, such as anti-angiogenic drugs or targeted mitochondrial therapies [12,13]. Additionally, these high-risk patients could undergo enhanced surveillance, including more frequent imaging studies.

Current research on predictive models for ovarian cancer resistance has largely focused on genomics and proteomics [14,15,16,17,18]. While promising, these molecular biomarkers are not yet widely validated for clinical use. Their high cost and complex detection requirements also limit their accessibility, particularly in community hospitals or resource-limited settings. By contrast, routine laboratory tests—such as complete blood counts, liver and kidney function panels, tumor markers, and coagulation profiles—are inexpensive, widely available, and routinely performed in clinical practice [15]. Previous studies have suggested a correlation between certain laboratory markers (such as the neutrophil-to-lymphocyte ratio and platelet-to-lymphocyte ratio) and platinum sensitivity [16,17]. The role of immune cells in tumorigenesis further supports the potential of these markers as valuable biomarkers [18].

Although individual laboratory markers show predictive potential, their accuracy is insufficient to guide clinical decisions alone. Artificial intelligence and machine learning are adept at analyzing high-dimensional, complex data and have proven to be effective tools for assessing chemotherapy response in ovarian cancer [19,20]. However, a dedicated model for predicting platinum resistance that relies exclusively on routine laboratory parameters is still lacking.

This study aims to systematically evaluate the predictive power of routine laboratory indicators for predicting platinum resistance in ovarian cancer, successfully establishing a clinical decision-support tool for early risk assessment. The main contributions of this work are threefold. First, regarding methodological innovation, we propose a novel Dynamic Weighted Fusion (DWF) model that employs a Softmax function with a low temperature coefficient to dynamically integrate multiple base learners, significantly enhancing prediction robustness compared to single classifiers. Second, in terms of biomarker discovery, we identify a synergistic “double-hit” signature driven by the interplay of coagulation and inflammation. Finally, demonstrating clinical utility, this cost-effective framework significantly outperforms the standard Logistic Regression (LR) benchmark. Crucially, it functions as a highly reliable bidirectional stratification tool; its superior negative predictive value effectively rules out resistance, granting clinicians the confidence to pursue standard platinum regimens, while its proactive risk alerts facilitate timely therapeutic optimization, particularly in non-omics contexts.

## 2. Materials and Methods

### 2.1. Study Population and Data Collection

This retrospective study involved patients diagnosed with ovarian cancer at the Department of Gynecologic Oncology, Fudan University Shanghai Cancer Center, between January 2019 and August 2023. Clinical and laboratory information was collected from the hospital’s electronic medical records.

Patients were included if they met the following criteria: (1) a histopathological diagnosis of ovarian cancer; (2) received standardized platinum-based combination chemotherapy; and (3) had complete laboratory data available at the time of admission.

Exclusion criteria were: (1) concurrent malignancies or pregnancy in the last 6 months; (2) follow-up data were incomplete; or (3) a PFI between 6 and 12 months.

**Figure 1:**
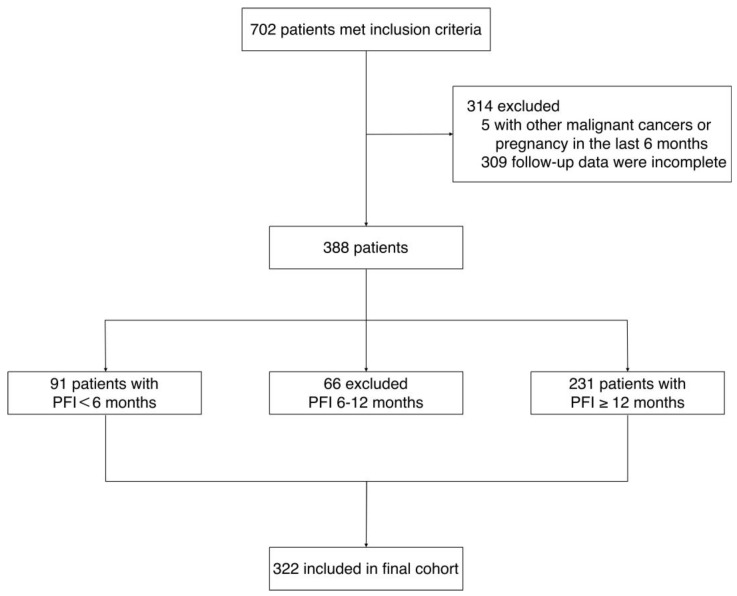
Flow diagram of patient selection.

The detailed patient selection process is illustrated in Figure 1.

### 2.2. Outcome Definition and Grouping

Patients were divided into two distinct groups based on their PFI: (1) Platinum-Resistant: Patients with a PFI of less than 6 months. This clinical endpoint serves as a proxy for intrinsic resistance, capturing the population with the most aggressive disease course and lack of therapeutic response. (2) Platinum-Sensitive: Patients with a PFI of 12 months or greater.

Patients with a PFI between 6 and 12 months, often defined as “partially platinum-sensitive”, were excluded from this study. This exclusion was a deliberate methodological choice to enhance the clarity of the machine learning model. These patients represent a clinically heterogeneous group with an intermediate response to platinum therapy [21], making them difficult for a binary classification algorithm to distinguish. By focusing on two well-defined, distinct endpoints, we aimed to improve the model’s ability to learn the defining features of clear resistance versus sustained sensitivity.

### 2.3. Candidate Features

For model construction, 70 candidate features were selected based on their universality in routine clinical use by gynecologic oncologists (Table 1). This feature set included 2 baseline clinical characteristics (Age and body mass index (BMI)) and 68 laboratory parameters obtained upon admission. The laboratory parameters were categorized into four main groups: 24 complete blood counts, 29 liver and renal function panels, 8 tumor markers, and 7 coagulation profiles.

### 2.4. Data Preprocessing

To ensure data quality, a sequential preprocessing pipeline was implemented:

1. Outlier Management: To mitigate the impact of extreme outliers and skewed distributions inherent in oncological laboratory data, we applied a truncation strategy based on clinical reference ranges. Specifically, values exceeding the standard upper limit were capped at that threshold. This approach was adopted because retaining the exact magnitude of extreme pathological values was found to introduce noise, thereby compromising model performance. By constraining these values, we enabled the model to focus on the critical transition from physiological homeostasis to pathological states, significantly enhancing the classifier’s robustness against outlier-driven bias.

2. Missing Value Imputation: A low proportion of missing data (approximately 1.62%) was addressed using Multivariate Imputation by Chained Equations [22]. This strategy was implemented with an IterativeImputer (30 maximum iterations). The Multivariate Imputation by Chained Equations algorithm operates by building a predictive model for each variable with missing values, using all other variables as predictors. This “chained” process iterates until the imputed values converge, effectively preserving the complex inter-variable correlations and avoiding the variance distortion often caused by simpler methods like mean or median imputation.

3. Feature Transformation: The Box–Cox transformation was applied to correct for skewed distributions observed in some features, such as tumor markers [23]. This power transformation method uses an optimized parameter (*λ*) to convert non-normal data into an approximately Gaussian distribution, which stabilizes variance and meets the assumptions of many machine learning algorithms [24].

4. Feature Scaling: All features were standardized to a [0, 1] range using Min-Max scaling. This step eliminates the influence of different measurement units and ensures that all features are comparable during model training, which is particularly important for distance-based algorithms.

### 2.5. Addressing Class Imbalance

The dataset exhibited a moderate class imbalance. To prevent the model from being biased towards the majority class, we employed the Borderline-SMOTE oversampling algorithm [25]. This method focuses on generating synthetic samples for minority class instances that lie near the decision boundary. Crucially, to rigorously prevent data leakage, this oversampling process was applied exclusively to the training folds within the cross-validation loop. The validation sets retained their original, real-world class distribution to ensure an unbiased evaluation of the model’s performance.

### 2.6. Construction of the DWF Model

**Figure 2:**
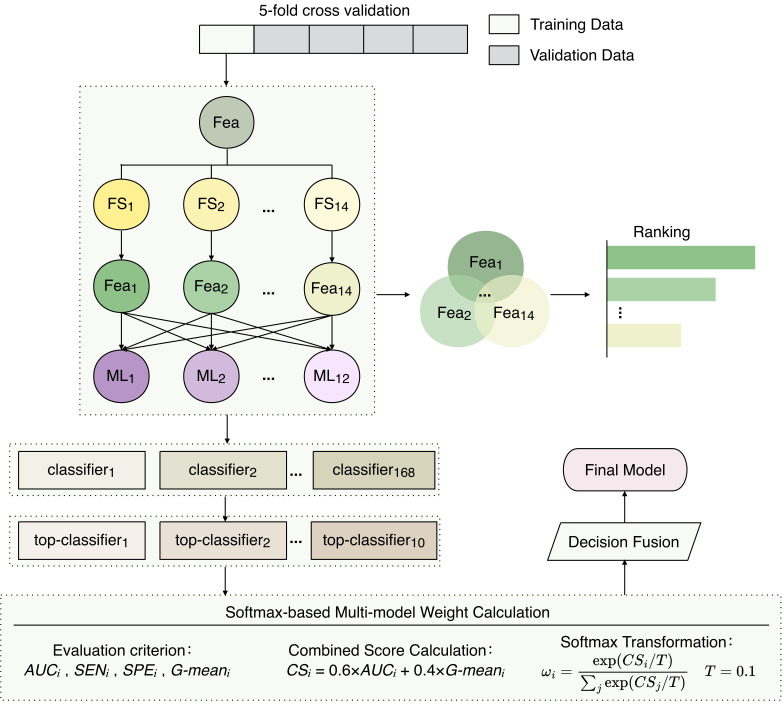
Workflow for DWF construction and validation.

To develop a high-performance risk prediction model, we established a DWF framework. This approach advances the multi-criteria fusion strategy validated by He et al. by incorporating specific optimizations [26]. To control overfitting and ensure robust generalization, we implemented a strict 5-fold nested cross-validation scheme. Specifically, we implemented a dynamic ensemble selection mechanism to identify the optimal base classifiers within each cross-validation fold. Furthermore, we prioritized the geometric mean (G-mean) for model screening to robustly address class imbalance. Finally, we employed a Softmax function with a low temperature coefficient to replace traditional distance-based weighting, thereby aggressively amplifying the influence of the highest-performing classifiers. The overall architecture and workflow of the proposed DWF framework are visually summarized in Figure 2. The specific implementation steps are as follows:

1. Nested Cross-Validation and Base Classifier Generation: We constructed a diverse ensemble of 168 base classifiers by combining 14 feature selection algorithms with 12 machine learning models (Table 2). To rigorously prevent information leakage, feature selection was performed exclusively on the training data of each fold. For each iteration, the top 10 features identified were used to train the 12 machine learning models. To capture a wide spectrum of data patterns—from simple linear correlations to complex non-linear biological interactions—we incorporated algorithms spanning four distinct modeling paradigms: (1) Linear Models (LR): effective for detecting direct linear relationships; (2) Distance-based Models (KNN and SVM): classifying patients via high-dimensional similarity; (3) Tree-based Models (DT, RF, Extra Trees, and Balanced RF): simulating clinical decision-making through hierarchical rules; and (4) Boosting Algorithms (AdaBoost, GradientBoosting, XGBoost, LightGBM, and CatBoost): advanced ensemble techniques that iteratively refine predictions to enhance accuracy. Crucially, to optimize hyperparameters and mitigate overfitting, we implemented an internal 3-fold cross-validation within each training set. As detailed in Table S1, hyperparameters were fine-tuned using Randomized Search strictly on the training data, ensuring the validation fold remained completely unseen during the optimization process.

2. Base Classifier Selection: Within each outer cross-validation fold, all 168 base classifiers were evaluated on the independent validation set using the G-mean as the primary performance metric. The G-mean is effective at assessing a model’s performance on both positive and negative classes in imbalanced datasets. The models were ranked by their G-mean score, and the top 10 were selected as the base learners for that fold’s ensemble.

3. Model Weighting and Fusion: To achieve an optimal fusion, a weight was calculated for each of the 10 selected base classifiers. This weight was derived from a combined score (*CS*) that incorporates both area under the curve (AUC) and G-mean. The score for the *i*-th base learner is defined as:

(1) $${CS}_{i}=0.6\cdot {\mathrm{AUC}}_{i}+0.4\cdot {G-\mathrm{mean}}_{i},$$

where ${\mathrm{AUC}}_{i}$ and ${G-\mathrm{mean}}_{i}$ are the respective metrics for the i-th model on the validation set.

4. Softmax Weight Allocation: We used a Softmax function with a temperature coefficient (*T*) to convert these scores into normalized weights. A low *T* value (e.g., 0.1) creates a sharper weight distribution, significantly amplifying the weight of the best-performing models while still retaining minor contributions from others. The final weight ($\omega_{i}$) for the *i*-th base learner was calculated as:

(2) $$\omega_{i}=\frac{exp({CS}_{i}/T)}{\sum_{j=1}^{10}\;exp({CS}_{j}/T)},$$

where *T* = 0.1.

5. Final Prediction: The final predictive probability of the DWF model ($P_{\mathrm{fusion}}$) is computed as the weighted average of the 10 selected base learners’ individual predictions $P_{i}$ (*i* = 1, …, 10).

(3) $$P_{\mathrm{fusion}}=\sum_{i=1}^{10}\;\omega_{i}\cdot P_{i}$$

6. Definition of Feature Importance Criteria: Beyond prediction, the framework also identifies important features based on their recurrence frequency. Specifically, we analyzed the feature subsets utilized by the top 10 base classifiers identified within each of the 5 cross-validation folds (totaling 50 optimized classifiers). The final importance ranking was determined by the accumulation frequency—i.e., how often a feature was prioritized by these 50 high-performing classifiers. Features with high recurrence frequencies represent the most robust predictors that consistently contribute to optimal classification performance across diverse data partitions.

### 2.7. Optimization of the Classification Threshold

The conventional classification threshold of 0.5 is often suboptimal for imbalanced datasets. To balance sensitivity and specificity, we identified an optimal threshold ($\theta_{\mathrm{opt}}$) by maximizing the G-mean. This was achieved by iterating through all possible thresholds on the receiver operating characteristic curve of the validation set and selecting the one that yielded the highest G-mean.

A patient was predicted as platinum-resistant if their fused risk score $P_{\mathrm{fusion}}$ exceeded the optimized threshold $\theta_{\mathrm{opt}}$; otherwise, they were predicted as platinum-sensitive. (Note: In the model framework, the platinum-resistant group is treated as the positive class.)

### 2.8. Model Performance Evaluation

Model performance was comprehensively evaluated using several metrics: AUC, accuracy, sensitivity, and specificity. The performance of our fusion model was also compared against both individual machine learning algorithms and single clinical predictors to demonstrate its superior accuracy and stability.

### 2.9. Feature Assessment

To elucidate intrinsic patterns, we calculated Spearman’s rank correlation coefficients and performed hierarchical clustering using Ward’s minimum variance method, visualizing the structure via a lower triangular heatmap. Finally, to ensure comprehensive model interpretability, we employed both odds ratio (OR) analysis and the Shapley additive explanations (SHAP) [27] framework.

### 2.10. Statistical Analysis

The Shapiro–Wilk test was employed to evaluate data distribution. Given that continuous variables exhibited a non-normal distribution, they are expressed as medians with interquartile ranges (IQR), except for the PFI, which is reported as the median with a 95% CI. Categorical variables are summarized as frequencies and percentages. For inter-group comparisons, the Mann–Whitney U test was applied to continuous variables, and the Chi-square test or Fisher’s exact test was utilized for categorical variables as appropriate. Survival curves were generated using the Kaplan–Meier method, and differences between groups were assessed using the log-rank test. All statistical tests were two-sided, with statistical significance defined as a *p*-value < 0.05. Data preprocessing, model construction, and statistical analyses were performed using R software (version 4.3.3; R Foundation for Statistical Computing, Vienna, Austria) and Python (version 3.6; Python Software Foundation, Wilmington, DE, USA).

## 3. Results

### 3.1. Baseline Characteristics

This study enrolled a total of 322 patients with ovarian cancer. Participants were stratified based on their PFI into either a platinum-resistant group (PFI < 6 months; n = 91) or a platinum-sensitive group (PFI ≥ 12 months; n = 231). This categorization resulted in an approximate 2:5 ratio between the platinum-resistant minority class and the platinum-sensitive majority class.

The baseline demographic, clinical, and laboratory characteristics of the study cohort are summarized in Table 3. The analysis revealed no statistically significant differences between the two groups regarding BMI (*p* = 0.916), histological subtype (*p* = 0.423), or the use of NAC (*p* = 0.189). This indicates that the resistant and sensitive cohorts were well-balanced in terms of nutritional status, tumor pathology, and initial treatment strategy. As anticipated by the study design, the median PFI differed significantly (38.5 vs. 3.0 months, *p* < 0.001).

Demographically, patients in the platinum-resistant group were significantly older than those in the sensitive group (61 vs. 55 years, *p* < 0.001). Regarding laboratory parameters, the resistant group exhibited signs of heightened inflammatory burden and metabolic activity, characterized by significantly elevated levels of the NL, GRAN, GRAN#, WBC, and UREA. Notably, for parameters such as LYM, FIB, and FDP, the median values appeared identical between groups, yet statistical analysis revealed significant differences. This finding reflects the properties of the rank-sum test, which detects shifts in the overall data distribution—such as differences in variance or skewness—rather than central tendency alone.

Collectively, these alterations reflect underlying imbalances in the inflammatory microenvironment and coagulation function in resistant patients, suggesting that these parameters may serve as valuable biomarkers for predicting platinum resistance and facilitating early risk stratification.

### 3.2. Survival Analysis

**Figure 3:**
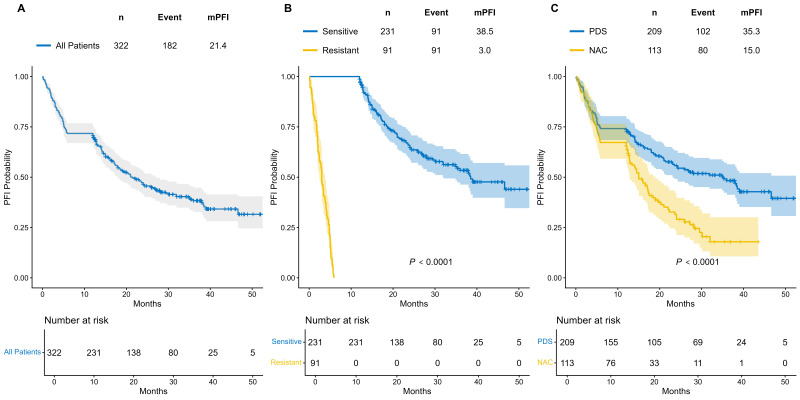
Kaplan–Meier curves for PFI (**A**) in the overall cohort; (**B**) stratified by platinum sensitivity; and (**C**) stratified by initial treatment strategy.

The Kaplan–Meier survival analysis was performed to evaluate the PFI across the entire cohort. As shown in Figure 3A, the median PFI for the total population was 21.4 months. We further stratified patients based on their clinical platinum status (Figure 3B). The analysis demonstrated a significant difference in survival outcomes between the two groups (*p* < 0.0001). The platinum-resistant group exhibited a rapid decline with a median PFI of 3.0 months. In contrast, the platinum-sensitive group showed a significantly prolonged survival profile with a median PFI of 38.5 months. Additionally, we compared PFI based on the primary treatment modality (Figure 3C). Patients who underwent PDS achieved a significantly longer median PFI of 35.3 months than those treated with NAC, who had a median PFI of 15.0 months (*p* < 0.0001).

### 3.3. Feature Engineering and Selection

#### 3.3.1. Feature Importance Ranking

**Figure 4:**
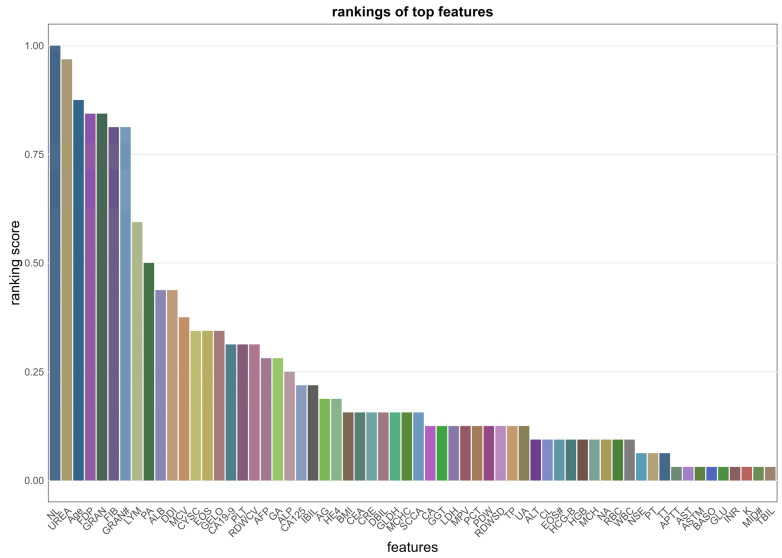
Feature importance ranking based on selection frequency. The ranking score reflects the frequency with which each feature was utilized by the top 10 base classifiers. The symbol ‘#’ indicates the absolute count of the respective blood cell populations. The full names of the laboratory items are listed in Table 1.

To identify the most robust predictive markers, we conducted an aggregated analysis of the feature subsets utilized by the top 10 base classifiers within each fold of the 5-fold cross-validation. From the initial pool of 70 candidate features, the union of subsets from these models yielded 62 unique features that were selected at least once. We then quantified the importance of each feature based on its selection frequency. As shown in Figure 4, the core determinants—specifically NL, UREA, Age, FDP, GRAN, FIB, GRAN#, LYM, PA, and ALB—emerged as the top 10 critical predictors. Notably, the top-tier features (e.g., NL, Age, FDP, FIB, GRAN) achieved a ranking score exceeding 0.80. This high cumulative frequency confirms that these drivers were consistently identified as critical predictors across individual folds, indicating minimal variance in feature importance.

#### 3.3.2. Feature Correlation Analysis

**Figure 5:**
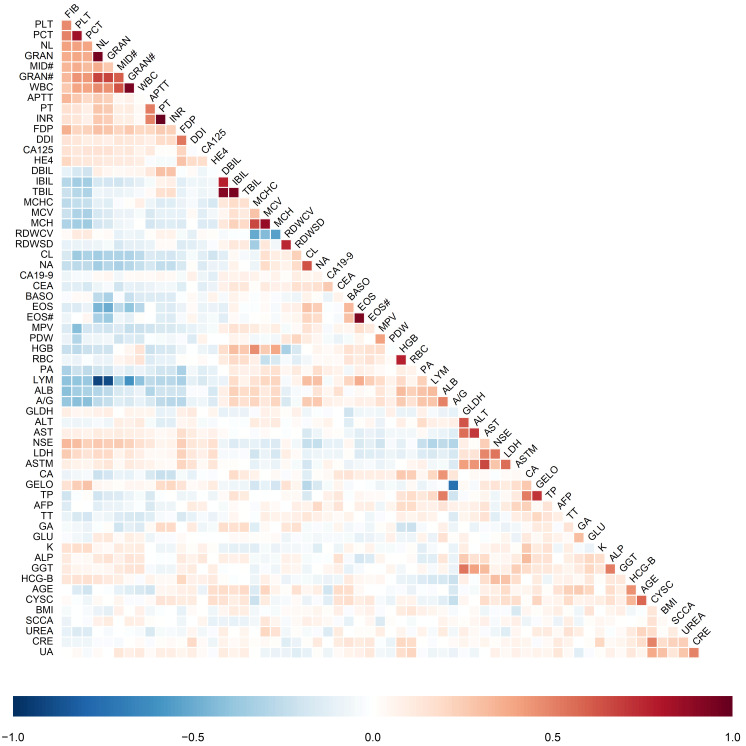
Spearman correlation coefficients between the included features. Features are reorganized based on hierarchical clustering. Red indicates positive correlation, and blue indicates negative correlation. The symbol ‘#’ indicates the absolute count of the respective blood cell populations. The full names of the laboratory items are listed in Table 1.

To elucidate the intrinsic physicochemical interplay and identify potential multicollinearity among the 62 candidate features, we performed a Spearman correlation analysis combined with hierarchical clustering (Figure 5). The resulting heatmap reveals distinct functional modules that mirror the systemic biological heterogeneity of ovarian cancer patients.

Notably, a strong positive correlation cluster was identified among hematological and inflammatory markers, specifically involving WBC, GRAN#, PLT, and FIB. This clustering signifies a synchronized systemic inflammatory response and hypercoagulable state, which are well-established hallmarks of the tumor microenvironment that promote stromal remodeling and impede platinum drug delivery. Furthermore, the inverse correlation observed between LYM and inflammatory indices highlights the potential prognostic value of NL in predicting chemotherapeutic response.

Distinct metabolic clusters were also observed: hepatic function indicators (TBIL, DBIL, IBIL, AST, ALT) and renal function markers (UREA, CRE, UA) formed independent, high-density blocks. Given that platinum-based agents are characterized by specific pharmacokinetic profiles and renal toxicity, these clusters reflect the patient’s baseline metabolic capacity to tolerate and clear cytotoxic agents, which indirectly influences dosing intensity and resistance development. Tumor markers (CA125, HE4) showed moderate correlations with specific biochemical parameters but remained relatively distinct, suggesting that traditional biomarkers alone capture only a fraction of the resistance variance. The complex collinearity and non-linear dependencies revealed in this analysis underscore the limitations of univariate analysis and justify the deployment of the high-dimensional machine learning feature fusion strategy utilized in this study.

#### 3.3.3. Univariable Performance

**Figure 6:**
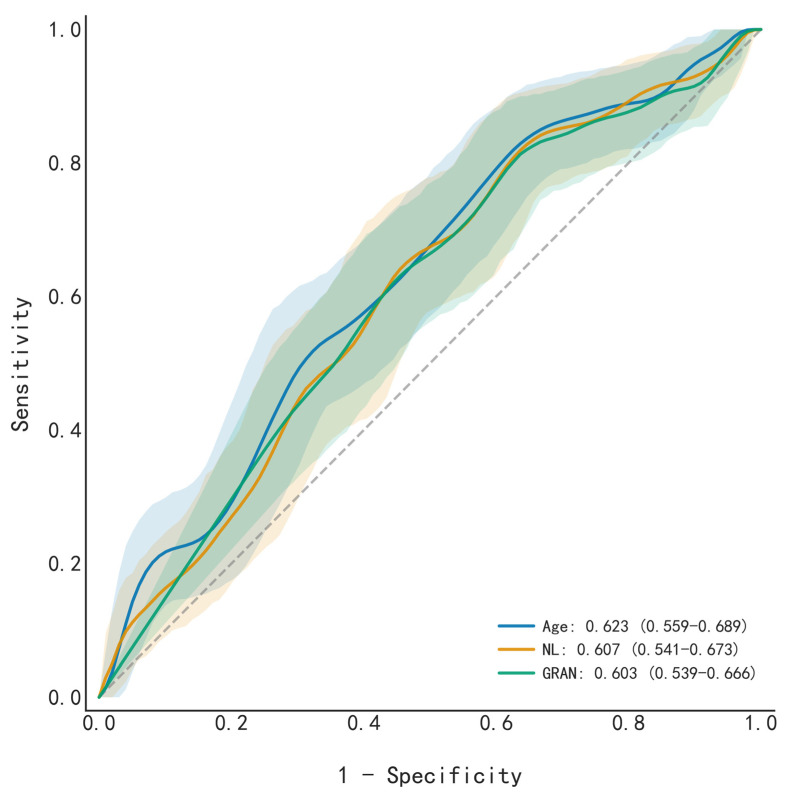
Receiver operating characteristic (ROC) curves for the top three predictors (Age, NL, and GRAN) of platinum resistance. The ROC curve of each feature was individually assessed using univariate logistic regression. The full names of the laboratory items are listed in Table 1.

Univariable logistic regression analysis was performed to assess the predictive value of all 62 features. Figure 6 highlights the top three performers: Age, NL, and GRAN, which yielded AUCs of 0.623, 0.607, and 0.603, respectively. However, the moderate predictive capability of even these leading indicators underscores the limitations of single biomarkers, thereby validating the necessity of a multivariable modeling approach.

### 3.4. Performance Evaluation of DWF

#### 3.4.1. Base Classifier Performance Evaluation

**Figure 7:**
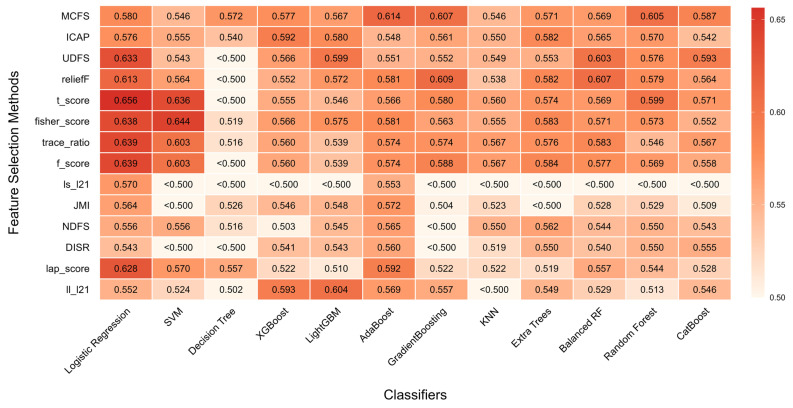
Predictive performances of base classifiers. The horizontal axis represents 12 machine learning models, while the vertical axis displays 14 feature selection methods. Each square in the grid represents a unique model, with its performance—measured by the AUC—indicated both numerically and visually through color.

To establish the need for an ensemble approach, we first evaluated the predictive efficacy of all single base classifiers (constructed from 14 feature selection methods and 12 machine learning models). The resulting AUC heatmap (Figure 7) showed that the combination of *T* score and Logistic regression yielded the best performance, yet its AUC was only 0.656. This confirmed the limitations of single-algorithm approaches.

#### 3.4.2. DWF Performance and Comparative Evaluation

**Figure 8:**
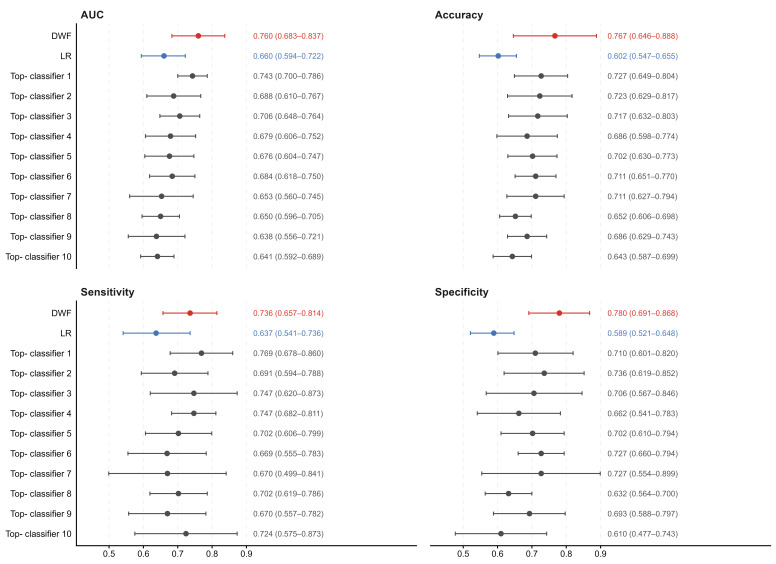
Performance evaluation of the DWF model versus the state-of-the-art LR benchmark and top base classifiers. The figure displays forest plots for four performance metrics: AUC, Accuracy, Sensitivity, and Specificity. Red represents the proposed DWF model, while blue represents the LR model. LR serves as the state-of-the-art benchmark; this baseline was constructed using the optimal single-classifier strategy (10 features selected via *T*-score) identified in Figure 7. Grey indicates the top 10 individual base classifiers. Central dots indicate mean values, and horizontal error bars represent 95% confidence intervals. Numerical values for the means and 95% CIs are presented to the right of each plot. DWF = dynamic weighted fusion model. LR = Logistic Regression. Top-classifier x = the individual base classifier ranked x-th based on predictive performance.

**Figure 9:**
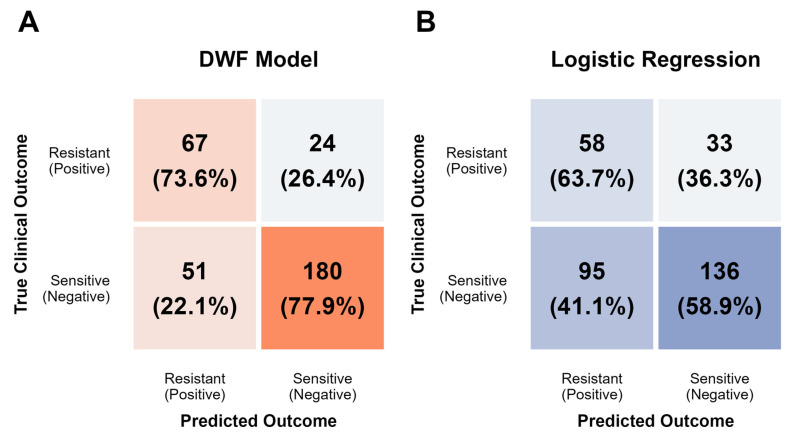
Pooled confusion matrix of the DWF model and the LR baseline. (**A**) Pooled confusion matrix for the proposed DWF model. (**B**) Pooled confusion matrix for the LR benchmark model.

To overcome the limitations of individual base classifiers, we constructed the DWF model. Figure 8 presents the performance metrics across five-fold cross-validation. The DWF model demonstrated robust predictive capability, achieving an AUC of 0.760 (95% CI: 0.683–0.837) and an accuracy of 0.767 (95% CI: 0.646–0.888). As detailed in the pooled confusion matrix (Figure 9A), the model exhibited a balanced classification profile, correctly stratifying 180 out of 231 sensitive cases and 67 out of 91 resistant cases. This translates to a high specificity of 0.780 and a sensitivity of 0.736, indicating that the DWF model effectively minimizes both false positives and false negatives.

Notably, the DWF model achieved numerically superior performance across all metrics compared to the top-ranked individual base classifier. Although the DeLong test indicated no statistically significant difference between the DWF model and the top classifier (*p* > 0.05), the consistent numerical improvement suggests that the fusion strategy successfully enhances model stability and generalization beyond any single algorithm.

To rigorously benchmark this performance, we compared the DWF model against the standard clinical methodology. LR serves as the state-of-the-art benchmark due to its widespread adoption in ovarian cancer resistance research; the baseline was constructed using 10 features selected via *T*-score, identified as the optimal single-classifier strategy in Figure 7. Despite using this optimized feature set, the LR baseline exhibited limited performance compared to the DWF model (Figure 8 and Figure 9B). The LR model achieved a significantly lower AUC of 0.660 (95% CI: 0.594–0.722). Most critically, the confusion matrix analysis reveals that the LR model struggles with specificity (0.589), correctly identifying only 136 sensitive cases compared to 180 by the DWF model. These results demonstrate that by integrating diverse base classifiers, the DWF framework captures complex non-linear associations that simple linear models miss, delivering more stable and clinically applicable performance.

#### 3.4.3. Prognostic Value and Risk Stratification

**Figure 10:**
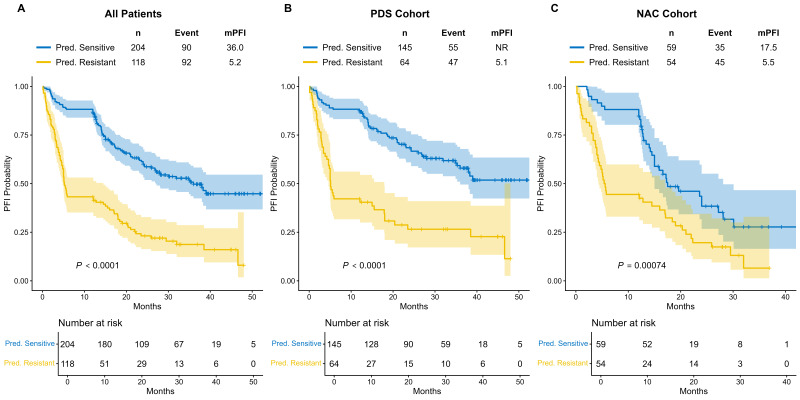
Kaplan–Meier survival analysis for PFI stratified by model-predicted labels. (**A**) Kaplan–Meier curves comparing the PFI between predicted sensitive and predicted resistant patients in the overall study cohort. (**B**) Survival analysis within the PDS subgroup. (**C**) Survival analysis within the NAC subgroup.

To further evaluate the prognostic significance of the model’s predictions, Kaplan–Meier survival analyses for PFI were performed across the entire study population and within the stratified treatment subgroups (Figure 10). In the overall cohort (Figure 10A), patients categorized as “Pred. Sensitive” demonstrated a significantly prolonged median PFI compared to those in the “Pred. Resistant” group (36.0 vs. 5.2 months, *p* < 0.0001).

Importantly, this robust risk stratification capability was maintained when patients were stratified by their initial treatment strategies. Within the PDS cohort (Figure 10B), the predicted sensitive group exhibited exceptional clinical outcomes, with the median PFI not reached (NR), whereas the predicted resistant group had a median PFI of merely 5.1 months (*p* < 0.0001). Similarly, in the NAC cohort (Figure 10C), the model effectively discriminated between distinct prognostic groups. The predicted sensitive patients in this cohort experienced a significantly longer median PFI than the predicted resistant patients (17.5 vs. 5.5 months, *p* = 0.00074). These findings collectively indicate that the model’s predictions serve as a strong and independent prognostic indicator for disease progression, regardless of the initial clinical intervention.

### 3.5. Model Subgroup Analysis and Interpretability

#### 3.5.1. Subgroup Analysis Based on Treatment Strategy

To further evaluate the robustness and clinical applicability of the DWF model, a stratified analysis was conducted based on the patients’ initial treatment strategies (Table 4). The model demonstrated remarkably consistent overall discriminatory performance across both subgroups, achieving an AUC of 0.755 (95% CI: 0.641–0.870) in the PDS cohort and 0.761 (95% CI: 0.659–0.864) in the NAC cohort.

Interestingly, the model exhibited distinct diagnostic profiles between the two treatment strategies. In the PDS group, the model yielded a higher overall accuracy (0.795, 95% CI: 0.643–0.946) and a notably superior specificity (0.829, 95% CI: 0.616–1.000) compared to the NAC group. Conversely, in the NAC cohort, the model demonstrated enhanced sensitivity (0.831, 95% CI: 0.662–1.000) relative to the PDS cohort (0.674, 95% CI: 0.552–0.795). Both subgroups maintained excellent negative predictive values (NPV), reaching 0.879 and 0.891 for the PDS and NAC groups, respectively, indicating the model’s strong reliability in identifying true negative cases. It is worth noting that the relatively wide confidence intervals observed in certain metrics are reflective of the reduced sample sizes inherent to the subgroup stratification.

#### 3.5.2. ORs Analysis of Included Features

**Figure 11:**
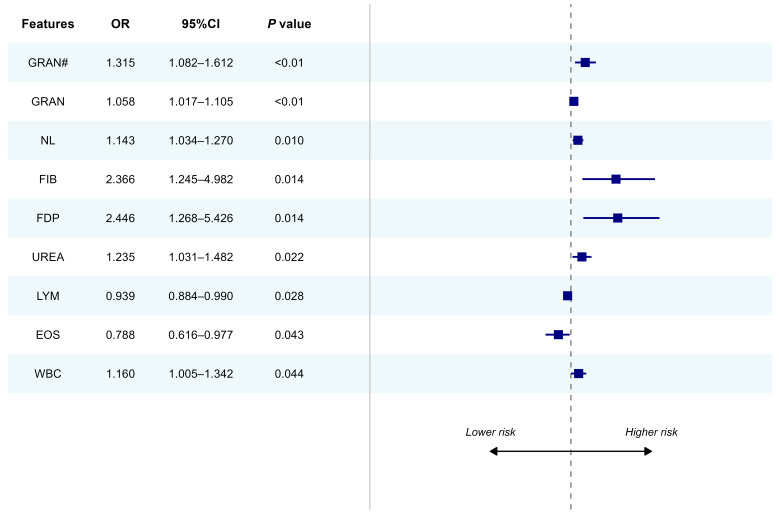
Univariate forest plot of significant features associated with platinum resistance. For each feature, the square represents the OR, and the corresponding horizontal line indicates the 95% CI. Note: This plot reflects individual feature associations. The symbol ‘#’ indicates the absolute count of the respective blood cell populations.

To further elucidate the independent impact of baseline parameters on the risk of platinum resistance, we analyzed their ORs. As illustrated in Figure 11, dysregulated coagulation was significantly associated with increased resistance risk. Specifically, FDP (OR = 2.446, *p* = 0.014) and FIB (OR = 2.366, *p* = 0.014) emerged as potent risk factors, indicating a strong statistical association between a hypercoagulable state and the clinical phenotype of resistance. Furthermore, an exacerbated systemic inflammatory burden—manifested by elevated GRAN# (OR = 1.315, *p* < 0.01), NL (OR = 1.143, *p* = 0.010), and WBC (OR = 1.160, *p* = 0.044)—constituted independent risk factors. UREA levels demonstrated a similar positive correlation (OR = 1.235, *p* = 0.022).

Conversely, higher LYM (OR = 0.939, *p* = 0.028) and EOS (OR = 0.788, *p* = 0.043) were identified as protective factors, suggesting that a robust immune profile may facilitate better therapeutic sensitivity. These findings align closely with the feature importance ranking derived from the DWF model, further validating the biological plausibility of the selected predictors.

#### 3.5.3. Model Interpretability Analysis Based on SHAP

**Figure 12:**
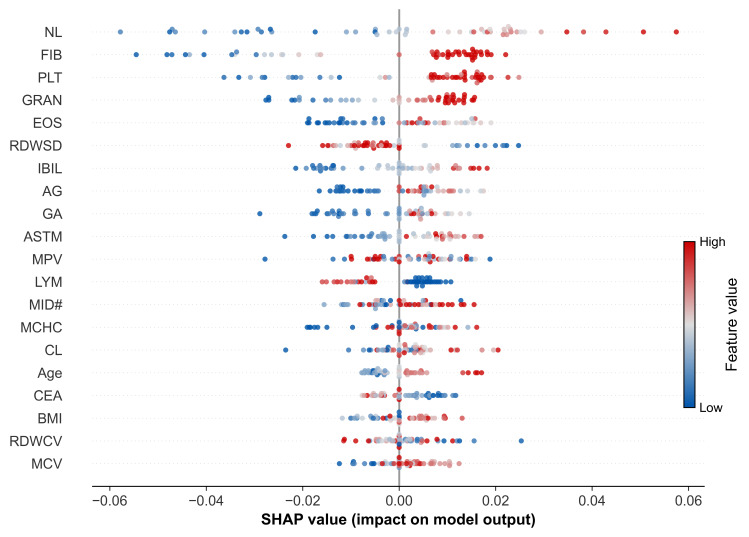
SHAP summary plot for the top 20 most important features. Features are ranked in descending order of importance. Each point represents a sample. The horizontal position indicates the SHAP value, where positive values push the prediction toward “resistant” and negative values push toward “sensitive”. The color represents the feature’s value, ranging from low (blue) to high (red). Note: This plot illustrates the top contributors to the model’s predictive accuracy. It includes features that contribute via non-linear interactions, even if not statistically significant in linear regression, while some high-risk features may be masked here due to collinearity. The symbol ‘#’ indicates the absolute count of the respective blood cell populations.

To elucidate the decision-making logic of the DWF model, we employed SHAP analysis to visualize feature contributions (Figure 12). The results indicated that the SHAP values for all features converged within a narrow range of −0.06 to 0.06. This distribution suggests that the model does not rely on a single dominant factor but rather makes decisions through the synergistic contribution of multiple biomarkers. Among the top-ranked features, elevated levels of NL, FIB, and PLT were associated with an increased probability of predicted resistance, highlighting the role of systemic inflammation and hypercoagulability. Conversely, the model identified immunosuppression as a critical risk factor, with lower levels of LYM contributing to a higher likelihood of resistance.

In summary, SHAP analysis not only quantified feature importance but also revealed the pathophysiological rationale underlying the model’s predictions: a comprehensive assessment integrating the patient’s inflammatory response, immune status, and coagulation dysfunction.

## 4. Discussion

Platinum resistance remains the critical bottleneck limiting survival benefits in ovarian cancer, yet reliable pre-treatment predictive tools remain elusive. Current clinical standards rely heavily on the retrospective assessment of the PFI. This lagging evaluation inevitably causes patients to miss the optimal window for personalized intervention. To address this gap, we developed a DWF model based on 60 routine laboratory indicators, alongside Age and BMI. To our knowledge, this is the first study to apply ensemble machine learning algorithms to the prediction of platinum resistance in ovarian cancer, successfully marking a paradigm shift from retrospective confirmation to prospective prediction.

Unlike existing models that depend on expensive genomics, radiomics, or features available only after surgery or chemotherapy (e.g., FIGO stage, residual disease size, or chemotherapy cycles), our model offers distinct advantages for widespread clinical implementation. First, all features are derived from standardized, low-cost pre-treatment admission data, effectively overcoming barriers to adoption in primary healthcare settings. Second, the model can function as a seamless decision-support module embedded within electronic health record systems.

The predictive power of the DWF model derives not from single biomarker abnormalities, but from a comprehensive quantification of the complex host-tumor interaction. The model identifies a high-risk phenotype characterized by a “double-hit” mechanism: a hypercoagulable state restricting drug delivery and systemic immune exhaustion limiting tumor clearance, which likely contributes to resistance through stromal remodeling rather than intrinsic cellular mutations.

Specifically, the synergistic elevations in FIB and FDP suggest a hypercoagulable microenvironment. Biologically, elevated fibrinogen facilitates the formation of a dense fibrin meshwork around tumor nests. This acts as a physical barrier that hinders the perfusion and diffusion of platinum agents, effectively preventing the drug from reaching therapeutic concentrations within the tumor [28]. Furthermore, fibrinogen serves as a provisional matrix that supports tumor cell adhesion and survival. This interaction creates a protective niche that shields tumor cells from chemotherapy-induced apoptosis, providing a survival advantage [29]. Therefore, while these systemic markers do not cause the genetic alterations typical of intrinsic resistance, they characterize a “physically shielded” microenvironment that creates a formidable barrier to therapeutic efficacy.

Complementing this physical barrier, the resistant group exhibited a distinct pattern of immune exhaustion, primarily quantified by the NL. The observed divergence between high systemic inflammation (GRAN#) and depleted host immune reserves (LYM) suggests that chronic inflammation is antagonizing anti-tumor immunity. Consequently, the NL serves as a robust metric for this critical imbalance. Indeed, an elevated NL signifies a systemic dysregulation associated with poor therapeutic response across various solid tumors [30]. The superior performance of the DWF model likely results from its ability to capture a high-risk phenotype characterized by the confluence of a hypercoagulable state, systemic inflammation, and compromised anti-tumor immunity, rather than relying on isolated markers.

SHAP analysis further deconstructed the model’s decision-making logic, revealing that individual features contributed limited distinct values (concentrated between −0.06 and 0.06). This distribution offers a critical clinical insight: platinum resistance is driven by the synergistic effect of multidimensional abnormalities rather than a single dominant factor. Ensemble learning algorithms effectively integrate these synergistic signals, which are often too complex for human cognition to process, explaining why the ensemble model significantly outperforms individual laboratory tests. However, comparing the SHAP feature importance with the model’s selection stability reveals a critical nuance regarding how the algorithm handles biological redundancy.

A distinct methodological advantage of the DWF model lies in its handling of multicollinearity, such as that observed among coagulation (FIB/FDP) and inflammation (GRAN/GRAN#) markers. While traditional linear models necessitate variable reduction to prevent instability, our ensemble architecture, driven primarily by tree-based algorithms, leverages these correlations to enhance robustness. We observed a “Risk-Contribution Divergence”: although all four markers achieved high selection stability (Ranking Score > 0.80, Figure 4), the SHAP analysis (Figure 12) exhibited a “masking effect” where predictive credit was primarily attributed to representative features (FIB and GRAN), obscuring their correlates (FDP and GRAN#). Crucially, this masking does not imply redundancy. As evidenced by the forest plot (Figure 11), the “masked” features actually demonstrated stronger intrinsic risk associations, with FDP (OR = 2.446) and GRAN# (OR = 1.315) surpassing their counterparts. By retaining these correlated features, the model effectively aggregates diverse decision boundaries—utilizing high-frequency features for stability while capturing the steeper risk gradients of high-magnitude markers—thereby avoiding the erroneous exclusion of potent risk factors.

Consequently, the decision to employ a complex ensemble architecture is justified by a substantial improvement in clinical utility that simple linear models cannot achieve. Our benchmarking revealed that the standard LR model, constrained by its inherent linear assumptions, yielded a specificity of 0.589. In contrast, the DWF model effectively mapped these complex decision boundaries, elevating overall specificity from 0.589 to 0.780. A detailed evaluation of predictive metrics indicates that the model’s primary clinical advantage lies in bidirectional stratification, particularly its robust capacity to safely rule out resistance. The model’s high overall NPV provides clinicians with confidence to continue standard platinum-based regimens without the risk of administering ineffective treatment. Conversely, although PPV is inherently limited by the relatively low prevalence of resistance, a “predicted resistant” flag can serve as an early-warning signal for clinical management, prompting closer surveillance and proactive therapeutic planning.

Building on the model’s overall performance, subgroup analyses revealed distinct diagnostic profiles under different initial treatment strategies. In both the NAC (NPV = 0.891) and PDS (NPV = 0.879) cohorts, patients predicted as “sensitive” are highly likely to be truly sensitive, indicating that the model reliably identifies chemosensitive cases across both groups. Despite this shared high NPV, the two cohorts exhibit different error patterns that shape their clinical utility. In the NAC cohort, high sensitivity (0.831) ensures that few truly resistant cases are missed, thereby minimizing the risk of under-treatment. As highlighted by Perrone et al. [31], extending NAC beyond the conventional six cycles can substantially increase the likelihood of achieving R0 resection, provided the tumor remains chemosensitive. Thus, for NAC patients predicted as sensitive, the model safely supports extending treatment cycles to further downstage the tumor. Conversely, in the PDS cohort, the model demonstrated higher specificity (0.829), indicating a lower false-positive rate for identifying resistance. While its PPV remains moderate (0.657)—meaning predictions of resistance should be interpreted with caution—these “high-risk” predictions still provide a valuable early-warning signal. Rather than definitively altering the standard of care, this signal can guide closer monitoring or prompt the consideration of early intensified strategies, such as incorporating bevacizumab—which has been shown to improve progression-free survival in high-risk groups [32]—or enrolling them in targeted clinical trials.

Despite these promising findings, our study has limitations. First, the retrospective design inevitably introduces selection bias. Second, given the single-center design, the generalizability of the DWF model to other healthcare systems requires further verification. While our rigorous internal nested cross-validation suggests robust performance, future research efforts should focus on multi-center external validation to confirm the model’s robustness and calibrate it for broader clinical application. Third, the current iteration relies solely on laboratory data; integrating multimodal data, such as radiomics or clinical text, could further enhance the detection of true resistant cases and improve the PPV. Finally, to establish a clear binary classification phenotype, we excluded patients with a PFI between 6 and 12 months. While this strategy was essential to maximize the signal-to-noise ratio for identifying core resistance-associated features during this initial model development phase, we acknowledge that it limits the model’s direct generalizability to this intermediate subgroup. In a clinical setting, these patients represent a “grey zone” requiring nuanced management. Addressing this limitation remains a critical objective for future research. Potential strategies may include recruiting larger multi-center cohorts to power multi-class prediction models or integrating longitudinal multimodal data to better characterize the heterogeneity within this intermediate population.

## 5. Conclusions

In conclusion, this study establishes a cost-effective and highly accessible ensemble machine learning framework for the pre-treatment prediction of platinum resistance in ovarian cancer. Demonstrating robust predictive capability (AUC: 0.760), the DWF model significantly outperforms the standard LR benchmark by addressing its limitation in specificity (improving from 0.589 to 0.780). Furthermore, the identification of a high-risk phenotype driven by hypercoagulability and immune exhaustion validates the model’s biological interpretability.

By synthesizing routine laboratory indicators with clinical demographics, the model provides a practical, evidence-based tool to assist clinicians in early therapeutic stratification. Importantly, this model serves not as a replacement for genomic precision medicine but as a complementary “pre-screening” tool. By identifying high-risk phenotypes characterized by stromal barriers, the DWF model facilitates early decision-making, particularly in resource-limited settings where advanced genomic testing is unavailable or delayed.

However, the translation of this model into clinical practice requires further rigorous validation. Future initiatives must prioritize external validation across diverse, multicenter cohorts to confirm the model’s robustness and generalizability. Additionally, the current reliance on tabular clinical data suggests a pathway for evolution; integrating multimodal data—including radiomics and genomics—could further enhance predictive precision. Ultimately, evolving this static pre-treatment assessment into a dynamic, real-time monitoring system represents a critical direction for advancing precision oncology in ovarian cancer care.

## Figures and Tables

**Table 1:** The 70 studied candidate features.

Item Category	Laboratory Items	Completeness
Clinical Characteristics	Age	100.00%
	BMI	100.00%
Complete Blood Count	White Blood Cell (WBC)	100.00%
	Lymphocyte (LYM)	100.00%
	Mid-range cells (MID)	100.00%
	Granulocyte (GRAN)	100.00%
	Eosinophil (EOS)	100.00%
	Basophil (BASO)	100.00%
	Lymphocyte Count (LYM#)	100.00%
	Mid-range cells Count (MID#)	100.00%
	Granulocyte Count (GRAN#)	100.00%
	Eosinophil Count (EOS#)	100.00%
	Basophil Count (BASO#)	100.00%
	Red Blood Cell (RBC)	100.00%
	Hemoglobin (HGB)	100.00%
	Hematocrit (HCT)	100.00%
	Mean Corpuscular Volume (MCV)	100.00%
	Mean Corpuscular Hemoglobin (MCH)	100.00%
	Mean Corpuscular Hemoglobin Concentration (MCHC)	100.00%
	Red Blood Cell Distribution Width—Coefficient of Variation (RDWCV)	100.00%
	Red Blood Cell Distribution Width—Standard Deviation (RDWSD)	100.00%
	Platelet (PLT)	100.00%
	Mean Platelet Volume (MPV)	98.76%
	Plateletcrit (PCT)	98.76%
	Platelet Distribution Width (PDW)	98.76%
	Neutrophil-to-Lymphocyte Ratio (NL)	100.00%
Liver/Renal Function	Total Bilirubin (TBIL)	100.00%
	Direct Bilirubin (DBIL)	100.00%
	Indirect Bilirubin (IBIL)	100.00%
	Alkaline Phosphatase (ALP)	100.00%
	Alanine Aminotransferase (ALT)	100.00%
	Aspartate Aminotransferase (AST)	100.00%
	AST/ALT Ratio (ASTM)	100.00%
	Lactate Dehydrogenase (LDH)	100.00%
	Gamma-Glutamyl Transferase (GGT)	100.00%
	Total Protein (TP)	100.00%
	Albumin (ALB)	100.00%
	Globulin (GELO)	100.00%
	Albumin/Globulin Ratio (A/G)	100.00%
	Non-Esterified Fatty Acids (NEFA)	100.00%
	Prealbumin (PA)	100.00%
	Glutamate Dehydrogenase (GLDH)	100.00%
	Cystatin C (CYSC)	97.52%
	Glycated Albumin (GA)	90.06%
	Urea (UREA)	100.00%
	Creatinine (CRE)	100.00%
	Uric Acid (UA)	100.00%
	Glucose (GLU)	97.83%
	Calcium (CA)	100.00%
	Phosphorus (P)	100.00%
	Magnesium (MG)	100.00%
	Potassium (K)	100.00%
	Sodium (NA)	100.00%
	Chloride (CL)	100.00%
	Total Carbon Dioxide (TCO2)	100.00%
Tumor Markers	Squamous Cell Carcinoma Antigen (SCCA)	95.96%
	Carbohydrate Antigen 19-9 (CA19-9)	100.00%
	Carbohydrate Antigen 125 (CA125)	100.00%
	Alpha-Fetoprotein (AFP)	98.45%
	Carcinoembryonic Antigen (CEA)	100.00%
	Neuron-Specific Enolase (NSE)	94.41%
	Human Chorionic Gonadotropin Beta-subunit (HCG-B)	87.89%
	Human Epididymis Protein 4 (HE4)	99.38%
Coagulation Profile	Prothrombin Time (PT)	91.30%
	Fibrinogen (FIB)	91.30%
	Activated Partial Thromboplastin Time (APTT)	91.30%
	Thrombin Time (TT)	91.30%
	International Normalized Ratio (INR)	91.30%
	D-Dimer (DDI)	90.68%
	Fibrinogen/Fibrin Degradation Products (FDP)	90.68%

Note: The symbol “#” denotes the absolute count of the respective blood cell populations.

**Table 2:** Feature selection methods and machine learning models used for base classifier construction.

Category	Method/Model
Feature selection methods	ANOVA *F*-value selection (*F* score)
	ANOVA *T*-value selection (*T* score)
	Double Input Symmetrical Relevance (DISR)
	Fisher score
	Interaction Capping (ICAP)
	Joint Mutual Information (JMI)
	Laplacian score
	Logistic Loss-based l_2,1-Norm Minimization (LL l21)
	Least Square Loss-based l_2,1-Norm Minimization (LS l21)
	Multi-Cluster Feature Selection (MCFS)
	Non-negative Discriminative Feature Selection (NFDS)
	Relief-F algorithm (reliefF)
	Trace ratio criterion (Trace ratio)
	Unsupervised Discriminative Feature Selection (UDFS)
Machine learning models	Adaptive Boosting classifier (AdaBoost)
	Balanced Random Forest (Balanced RF)
	Categorical Boosting classifier (CatBoost)
	Decision Tree (DT)
	Extremely randomized trees (Extra Trees)
	Gradient-Boosting Machine (Gradient-Boosting)
	K-Nearest Neighboring classifier (KNN)
	Light Gradient-Boosting Machine (LGBM)
	Logistic Regression (LR)
	Random Forest (RF)
	Support Vector Machine (SVM)
	eXtreme Gradient-Boosting machine (XGBoost)

**Table 3:** Baseline characteristics of the study population based on platinum sensitivity status.

Characteristics	Platinum-Sensitive (231)	Platinum-Resistant (91)	*p*
Demographics			
Age (years)	55.00 (47.00–63.00)	61.00 (52.00–65.00)	<0.001
BMI (kg/m^2^)	23.23 (21.56–25.24)	23.44 (21.83, 25.04)	0.916
Histology, n (%)			0.423
High-grade Serous	177 (79.4%)	75 (83.3%)	
Others	46 (20.6%)	15 (16.7%)	
NAC, n (%)			0.189
Yes	76 (32.9%)	37 (40.7%)	
No	155 (67.1%)	54 (59.3%)	
Clinical Outcome			
Median PFI (months)	38.50 (32.00-NR)	3.00 (2.40–3.80)	<0.001
Laboratory Markers			
NL	3.57 (2.42–5.09)	4.43 (3.11–5.80)	0.003
GRAN (%)	70.80 (64.10–75.00)	74.00 (68.40–75.00)	0.003
GRAN # (×10^9^/L)	4.60 (3.60–6.30)	5.10 (4.40–6.30)	0.007
LYM (%)	20.00 (20.00–26.60)	20.00 (20.00–22.20)	0.008
FIB (g/L)	4.00 (3.52–4.00)	4.00 (3.93–4.00)	0.009
FDP (μg/mL)	5.00 (5.00–5.00)	5.00 (5.00–5.00)	0.010
UREA (mmol/L)	4.08 (3.33–5.00)	4.51 (3.41–5.61)	0.024
WBC (×10^9^/L)	6.60 (5.50–8.40)	7.10 (6.20–8.60)	0.034

Note: Data are presented as n (%) or median (IQR), except for median PFI, which is presented as median (95% CI). Comparisons were performed using the Mann–Whitney U test for continuous variables, the Chi-square test or Fisher’s exact test for categorical variables, and the Log-rank test for PFI. Data on histology were available for 313 patients (9 missing). BMI = body mass index. NAC = neoadjuvant chemotherapy. PFI = platinum-free interval. NR = Not Reached. The symbol “#” denotes the absolute count of the respective blood cell populations. The full names of the laboratory items are listed in Table 1.

**Table 4:** Performance of the DWF model stratified by initial treatment strategy.

	AUC	Accuracy	Sensitivity	Specificity	PPV	NPV
PDS	0.755 (0.641–0.870)	0.795 (0.643–0.946)	0.674 (0.552–0.795)	0.829 (0.616–1.000)	0.657 (0.326–0.988)	0.879 (0.835–0.923)
NAC	0.761 (0.659–0.864)	0.720 (0.626–0.815)	0.831 (0.662–1.000)	0.678 (0.498–0.858)	0.572 (0.372–0.771)	0.891 (0.806–0.977)

Note: Data are presented as means (95% CI).

## Data Availability

The data presented in this study are available upon request from the corresponding author. The data are not publicly available due to privacy and ethical restrictions involving patient information from the Shanghai Ovarian Cancer Specialty Database.

## Supplementary Materials

The following supporting information can be downloaded at: https://www.mdpi.com/article/10.3390/cancers18081190/s1, Table S1: Configuration and Tuning Space of Machine Learning Algorithms.

## Abbreviations

The following abbreviations are used in this manuscript:

DWF	Dynamic weighted fusion model
AUC	Area under the curve
PDS	Primary debulking surgery
NAC	Neoadjuvant chemotherapy
PFI	Platinum-free interval
BMI	Body mass index
G-mean	Geometric mean
CS	Combined score
OR	Odds ratio
IQR	Interquartile range
ROC	Receiver operating characteristic
CI	Confidence interval
vs	versus
SHAP	Shapley additive explanations
R0	No residual tumor
FIB	Fibrinogen
FDP	Fibrinogen/fibrin degradation products
GRAN	Granulocyte
NL	Neutrophil-to-lymphocyte ratio
LYM	Lymphocyte
WBC	White blood cell
EOS	Eosinophil
GRAN#	Granulocyte count
PLT	Platelet
TBIL	Total bilirubin
DBIL	Direct bilirubin
IBIL	Indirect bilirubin
AST	Aspartate aminotransferase
ALT	Alanine aminotransferase
CRE	Creatinine
UA	Uric acid
ALB	Albumin
PA	Prealbumin
CA125	Carbohydrate antigen 125
HE4	Human epididymis protein 4
NR	Not reached
NPV	Negative predictive values
PPV	Positive predictive values
