# Supplementary material for: Ensemble Machine Learning Predicts Platinum Resistance in Ovarian Cancer Using Laboratory Data
Source: Cancers (Basel). 2026 Apr 8;18(8):1190. doi: 10.3390/cancers18081190 (PMC13115104; doi:10.3390/cancers18081190)
Supplement: Supplementary file 1 [file cancers-18-01190-s001.zip › cancers-4109426-supplementary.pdf]

Supplementary Material

Article Title: Ensemble Machine Learning Predicts Platinum Resistance in Ovarian Cancer Using Laboratory Data

Authors: Xueting Peng [1], Yangyang Zhang [2], Chaoyu Zhu [3], Weijie Chen [3], Xiaohua Wu [2], Fan Zhong [3], Qinhao Guo [2,*] and Lei Liu [1 3 4,*]

Affiliation:

[1] Shanghai Institute of Infectious Disease and Biosecurity, Fudan University, Shanghai, China; 23211020027@m.fudan.edu.cn

[2] Department of Gynecologic Oncology, Fudan University Shanghai Cancer Center, Shanghai and China; 21301050051@m.fudan.edu.cn (Y.Z.); wu.xh@fudan.edu.cn (X.W.); 18111230017@fudan.edu.cn (Q.G.)

[3] Intelligent Medicine Institute, Fudan University, Shanghai, China; cyzhu18@fudan.edu.cn (C.Z.); wei-jie_chen@fudan.edu.cn (W.C.); zhongfan@fudan.edu.cn (F.Z.)

[4] Shanghai Institute of Stem Cell Research and Clinical Translation, Shanghai, China; liulei@fudan.edu.cn

[*] Correspondence: 18111230017@fudan.edu.cn (Q.G.); liulei@fudan.edu.cn (L.L.)

Table of Contents

Table S1. Configuration and Tuning Space of Machine Learning Algorithms

Model	Key Fixed Settings	Hyperparameter Search Space
Logistic Regression	class_weight='balanced'	C: [0.01, 0.1, 1, 10, 100]
KNN	weights='distance'	n_neighbors: [3, 5, 7, 9]
SVM	class_weight='balanced', probability=True	C: [0.1, 1, 10]<br>gamma: ['scale', 'auto', 0.01, 0.1]
Decision Tree	class_weight='balanced'	max_depth: [None, 5, 10, 15]
Extra Trees	class_weight='balanced'	n_estimators: [50, 100]<br>max_depth: [None, 10, 20]
Random Forest	class_weight='balanced'	n_estimators: [50, 100]<br>max_depth: [None, 10, 20]
Balanced RF	sampling_strategy='auto'	n_estimators: [50, 100]<br>max_depth: [None, 10, 20]
AdaBoost	algorithm='SAMME.R'	n_estimators: [50, 100]<br>learning_rate: [0.01, 0.1, 1.0]
Gradient Boosting	loss='log_loss'	n_estimators: [50, 100]<br>learning_rate: [0.01, 0.1, 1.0]
XGBoost	scale_pos_weight=3.2	n_estimators: [50, 100]<br>learning_rate: [0.01, 0.1, 0.3]<br>max_depth: [3, 5, 7]
LightGBM	class_weight='balanced'	n_estimators: [50, 100]<br>learning_rate: [0.01, 0.1, 0.3]<br>max_depth: [3, 5, 7]
CatBoost	auto_class_weights='Balanced'	iterations: [50, 100]<br>learning_rate: [0.01, 0.1, 0.3]<br>depth: [3, 5, 7]

Note: All models were initialized with a fixed random_state=42 for reproducibility. Fine-tuning was performed using a nested internal 3-fold cross-validation (cv=3) on the training set with 5 iterations (n_iter=5) per fold to identify optimal hyperparameters before final model training. For tree-based models, max_depth=None implies nodes are expanded until leaves are pure.
